# CRISPR/Cas9-induced transgene insertion and telomere-associated truncation of a single human chromosome for chromosome engineering in CHO and A9 cells

**DOI:** 10.1038/s41598-017-10418-7

**Published:** 2017-10-06

**Authors:** Narumi Uno, Kei Hiramatsu, Katsuhiro Uno, Shinya Komoto, Yasuhiro Kazuki, Mitsuo Oshimura

**Affiliations:** 1 Department of Biomedical Science, Institute of Regenerative Medicine and Biofunction, Graduate School of Medical Science, Tottori University, 86 Nishi-cho, Yonago, Tottori 683-8503 Japan; 2 Chromosome Engineering Research Center, Tottori University, 86 Nishi-cho, Yonago, Tottori 683-8503 Japan; 3 Department of Molecular and Cellular Biology, Faculty of Medicine, Tottori University, 86 Nishi-cho, Yonago, Tottori 683-8503 Japan

## Abstract

Chromosome engineering techniques including gene insertion, telomere-associated truncation and microcell-mediated chromosome transfer (MMCT) are powerful tools for generation of humanised model animal, containing megabase-sized genomic fragments. However, these techniques require two cell lines: homologous recombination (HR)-proficient DT40 cells for chromosome modification, and CHO cells for transfer to recipient cells. Here we show an improved technique using a combination of CRISPR/Cas9-induced HR in CHO and mouse A9 cells without DT40 cells following MMCT to recipient cells. Transgene insertion was performed in CHO cells with the insertion of enhanced green fluorescence protein (EGFP) using CRISPR/Cas9 and a circular targeting vector containing two 3 kb HR arms. Telomere-associated truncation was performed in CHO cells using CRISPR/Cas9 and a linearised truncation vector containing a single 7 kb HR arm at the 5′ end, a 1 kb artificial telomere at the 3′ end. At least 11% and 6% of the targeting efficiency were achieved for transgene insertion and telomere-associated truncation, respectively. The transgene insertion was also confirmed in A9 cells (29%). The modified chromosomes were transferrable to other cells. Thus, this CHO and A9 cell-mediated chromosome engineering using the CRISPR/Cas9 for direct transfer of the modified chromosome is a rapid technique that will facilitate chromosome manipulation.

## Introduction

**Figure 1:**
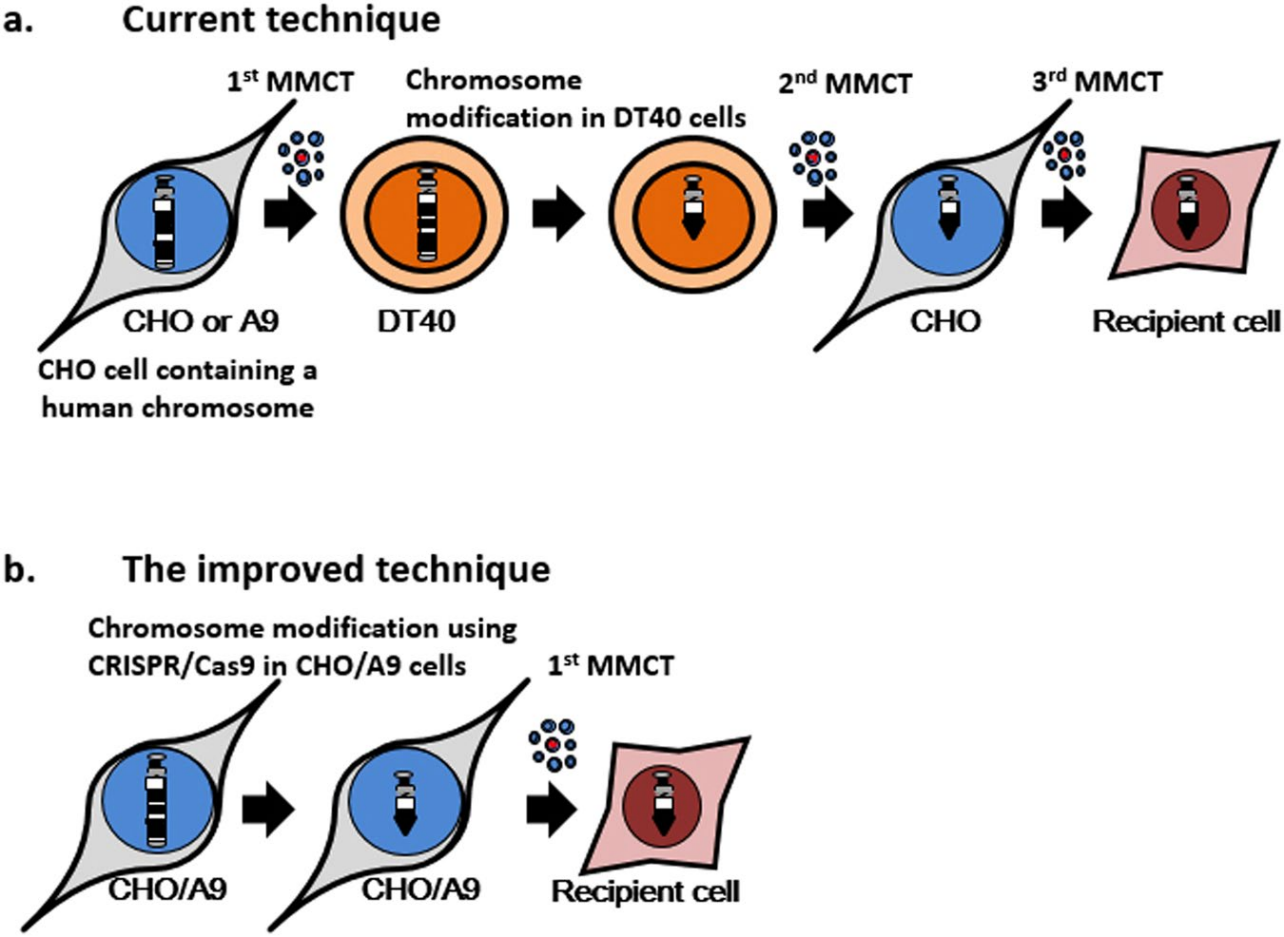
Schematic outline of chromosome engineering technique, including transgene insertion and telomere-associated truncation of a human chromosome 21 in CHO-K1 cells and the transgene insertion of a human chromosome 8 in mouse A9 cells using CRISPR/Cas9. (**a**) Outline of a current chromosome engineering technique using DT40 cells. This technique requires three rounds of MMCT to transfer a human chromosome to DT40 cells and re-transfer the modified chromosome to CHO cells. The modified chromosome can then be transferred to recipient cells. (**b**) Outline of the improved technique using CRISPR/Cas9 requires one round of MMCT to directly transfer the modified chromosome to recipient cells.

Chromosome engineering techniques using homologous recombination (HR)-proficient DT40 (chicken pre-B cells) efficiently induce transgene insertion and telomere-associated truncation via artificial telomere seeding^1^. The modified chromosomes can be transferred to CHO-K1 (Chinese hamster ovary-K1) cell line^1,2^ and to various other cell lines by microcell-mediated chromosome transfer (MMCT)^3^ (Fig. 1a). These transfers include to mouse embryonic stem cells for the generation of trans-chromosomic mice^4,5^, human embryonic stem cells to engineer aneuploid disease models^6^, pluripotent stem cells from patients with genetic disease for the genetic correction^7^ and cancer cell lines to investigate cancer suppressor genes^3,8,9^. In addition, mammalian artificial chromosome vectors, including human artificial chromosome (HAC)^10^ and mouse artificial chromosome (MAC)^11,12^ vectors into which megabase-sized chromosomal loci and multiple cDNAs can be loaded^13–17^, have been used with these techniques and cells. Such techniques are promising for various biomedical challenges. However, a modified chromosome in DT40 cells cannot be easily transferred into another cell type, except for into CHO-K1 cells. Therefore, the current technique requires three rounds of MMCT and repeated chromosomal analysis after each transfer (Fig. 1a).

Genome editing techniques using artificial nucleases (ZFNs^18^, TALENs^19^, CRISPR/Cas9^20^) have high HR efficiency of transgene insertion and deletion via targeted sequence-specific DNA breakage and DNA repair pathways in various host cells, even in fertilised eggs and somatic cells of various eukaryotic animals^21^. Recently, a CHO-K1 cell sub-strain, CHO-S, which is generally used for recombinant protein production, was modified with genome editing techniques^22–25^. Here, we report a direct manipulation of a specific transgene insertion and telomere-associated truncation in a single human chromosome in CHO-K1 cells using CRISPR/Cas9. The transgene insertion were also performed in mouse A9 cells containing a human chromosome 8 (hChr.8)^26^. In addition, MMCT of the modified chromosome was achieved from the CHO-K1 cells to recipient cells (Fig. 1b).

## Results

### Design of CRISPR/Cas9 and targeting plasmid vectors

Previously, we established CHO-K1 cells containing a human chromosome 21 (hChr.21)^10^. In this study, this hChr.21 was used for transgene insertion, telomere-associated truncation and transfer of the modified chromosome to other cells by MMCT.

**Figure 2:**
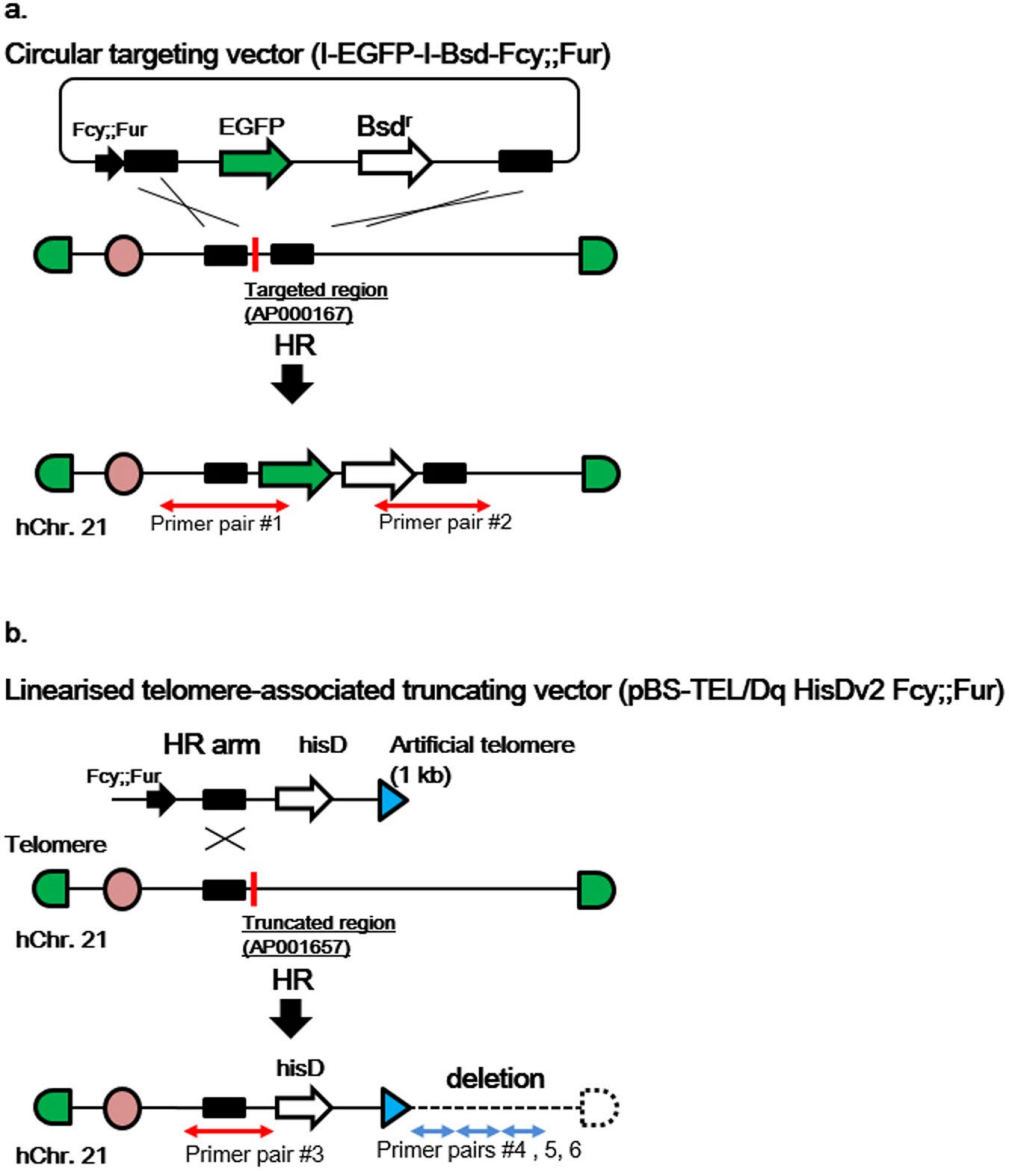
Schematic outline of gene-targeting and telomere truncation of human chromosome 21 in CHO-K1 cells using CRISPR/Cas9. (**a**) Human chromosome 21 was truncated in the region of AP000167 with CRISPR/Cas9, and the circular I-EGFP-I-Bsd Fcy; Fur vector inserted, which contained two homologous recombination arms, the EGFP gene and the blasticidin-resistance gene. Primer pair #1 and 2 were designed to detect the occurrence of homologous recombination. (**b**) Human chromosome 21 was truncated in the region of AP001657 with CRISPR/Cas9, and a linearised pBS-TEL/Dq HisDv2 Fcy; Fur vector inserted, which contained a single homologous recombination arm, 1 kb of artificial telomere sequence and a histidinol dihydrochloride-resistant gene (hisD). Primer pair #3 was designed to detect correct homologous recombination and primer pairs #4, 5 and 6 were designed to detect deletion of chromosome regions. Ideally, primer pair #3 should produce a PCR amplicon and primer pairs #4, 5, and 6 should not produce any PCR amplicons.

The targeting plasmid and telomere-associated truncation plasmid vectors contained homologous recombination (HR) arms, located near the centromere, and a positive selection resistance gene against blasticidin S (Bsd) or L-histidinol dihydrochloride (hisD). A suicide gene, Fcy; Fur, for negative selection, [Fcy; Fur causes cytotoxicity by metabolising 5-flucytosine (5-FC)] was inserted into the targeting plasmid vectors to eliminate cells harbouring unexpected insertion/s of a targeting plasmid vector in a host CHO chromosome (Fig. 2a and b).

To induce transgene insertion and telomere-associated truncation by homologous recombination at targeted sites, we designed sgRNA expressing plasmid vectors based on pX330, which can co-express sgRNA and SpCas9^27^.

AP000167 and AP001657 loci were targeted by CRISPR/Cas9. Two HR arms were randomly selected from the AP000167 locus. Candidate sgRNA sequences were selected from an approximately1kb region between the two HR arms using Optimized CRISPR design tool (http://crispr.mit.edu/)^27^. Similarly, the HR arm for telomere-associated truncation was randomly selected from the AP001657 locus. The candidate sgRNA sequences were selected from the region downstream of the selected HR arm. Six and four sgRNA-expressing pX330 vectors were designed for transgene insertion and telomere-associated truncation, respectively. These sgRNA-expressing vectors were then evaluated for their cleavage activity by Cel-I assays. The two vectors with the best cleavage activity were used for the subsequent experiments. These were AP000167T1 (cleavage activity: 17.2%) and T11(9.6%) for transgene insertion and AP001657T5 (7.4%) and T6 (9.4%) for telomere-associated truncation. Because, dual expression of two sgRNAs improves cleavage activity with the CRISPR/Cas9 system^28^, this chromosome modification in CHO-K1 cells has the advantage of reducing the possibility of CRISPR/Cas9 off-target activity. Generally, there is a possibility of off-target activity towards unexpected regions because of similar DNA sequences in other regions of the genome. This chromosome modification and transfer technique can target only one chromosome and even if the host chromosome was unexpectedly disrupted by CRISPR/Cas9, such off-target modifications of the host genome are excluded via MMCT. Therefore, choosing a specific sequence on one chromosome will reduce off-target events.

### Chromosomal transgene insertion and telomere-associated truncation using the CRISPR/Cas9 system in CHO-K1 cells

**Figure 3:**
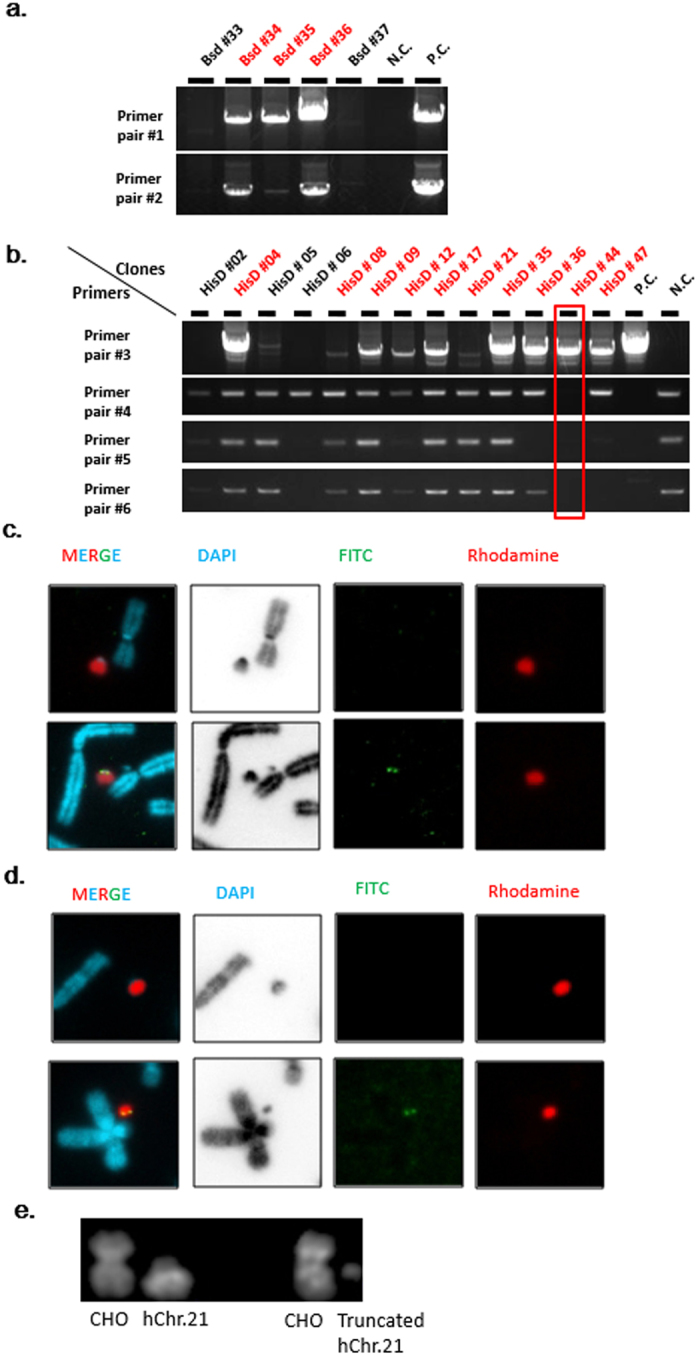
Representative PCR and FISH results of CHO cells containing the modified hChr.21. (**a**) Representative results of transgene insertion analysed by PCR (red for positive clones). The negative control (N.C.) was CHO (hChr.21), which contained intact hChr.21. The positive control (P.C) was a CHO clone containing 21HAC2, targeted with I-EGFP-I-Bsd in DT40 cells, as previously described^10^. (**b**) Representative results of telomere-associated truncation analysed by PCR (red for positive clones with primer pair #3). N.C. was CHO (hChr.21) and P.C. was a CHO clone containing 21HAC2. The red boxed lane shows the clone showing an ideal result (HisD #44). (**c**) Representative FISH images of transgene insertion. Panels on upper lane show an intact hChr.21 in CHO cell. Panels on under lane show a hChr.21 with the inserted transgene in CHO Bsd #36. The rhodamine (red) signal indicates the repetitive sequence of human Cot-1 for staining of whole human chromosome. The FITC signal (green), which was observed as a yellow dot, indicates the presence of the inserted I-EGFP-I-Bsd-Fcy; Fur vector on the near centromeric region. The chromosome size was similar between the intact hChr.21 and the transgene-inserted hChr.21. (**d**) Representative FISH images of telomere-associated truncation. The upper panels show an intact hChr.21 in CHO cell. The under panels show a truncated hChr.21 in CHO HisD #44. The rhodamine (red) signal indicates the human Cot-1 for staining of whole human chromosome. The FITC signal (green) indicates the presence of pBS-TEL/Dq-HisD vector in the terminal end of the truncated hChr.21. The chromosome size of the truncated hChr.21 in CHO HisD #44 was much smaller than the intact hChr.21. (**e**) Representative images of quinacrine hoechst staining. Left pair: a smallest CHO chromosome and a morphologically intact hChr.21 in CHO (hChr.21), right pair: a smallest CHO chromosome and a truncated hChr.21 in CHO HisD #44. The deletion of q11-terminal end was observed on the truncated hChr.21. Full-length gels of the PCR analysis are presented in Supplementary Figure 1.

To insert the targeting vector into hChr.21, the CHO-K1 cells containing hChr.21 were transfected with the two CRISPR/Cas9 vectors; AP000167T1 and T11 and the circular I-EGFP-I-Bsd-Fcy; Fur plasmid vector. The transfected cells were expanded for 5days and then treated with 8 µg/mL blasticidin S and 200 µM 5-FC (the substrate of Fcy; Fur) for 21 days. From the resulting colonies, 37 were randomly isolated, and genomic DNA was extracted. PCR analyses were performed with two primer pairs (primer pairs#1 and #2) (Fig. 2a) and 17 clones (46%) showed the expected HR bands (Fig. 3a) (see Supplementary Fig. 1a). Thirteen well growing clones were analysed by fluorescence *in situ* hybridisation (FISH), all the clones contained hChr.21 with various frequency (Table 1 and Fig. 3c). Thus, at least 13 (35%) of 37 isolated clones showed the transgene insertion. CHO cells are chromosomally abnormal, and often unstable with aneuploidy and polyploidy. Thus, the variation of the copy number of modified chromosome and polyploidy were likely occurred after the modification. Although it is not excluded that CRISPR/Cas9 induces chromosome instability, there are no report on this event.

**Table 1:** Summary of FISH analysis of CHO clones containing the targeted chromosome.

Ploidy	2n			4n		
Number of maintained hChr. 21	0	1	2	0	1	2
CHO Bsd#02	1	19				
CHO Bsd#06	1	17				1
CHO Bsd#16	1	19(del3)				
CHO Bsd#20	14	4(t2)		1		1
CHO Bsd#22		13	1			5
CHO Bsd#23	1	2			1(t1)	16(t10)
CHO Bsd#27		14(t1)	4	1		1(t1)
CHO Bsd#28	11	9(t1)				
CHO Bsd#30		16				4
CHO Bsd#32	4	14				2
CHO Bsd#34	18	1				1
CHO Bsd#35	3	12		1	1	3(del2)
CHO Bsd#36	1	17				1

Each column shows the number of cells in metaphase. The ploidy of CHO host chromosomes was 2 n or 4 n. The numbers of maintained hChr. 21 are shown in the second row. The number of cells showing hChr.21 aberration is indicated. del: deletion, t: translocation. In CHO Bsd #16, 19 cells contained a single hChr. 21, but 3 of 19 showed deletion of the chromosome.

To truncate a telomere of hChr.21 in CHO-K1 cells, the CHO-K1 cells containing hChr.21 were transfected with two CRISPR/Cas9 vectors, AP001657T5 and T6, and with the linearised truncating vector, pBS-TEL/Dq HisDv2-Fcy; Fur. The transfected cells were expanded for 5days and then treated with antibiotics (7 mM L-histidinol dihydrochrolid and 200 µM 5-FC). Forty-eight clones were isolated, and PCR analyses were performed with primer pair #3 (Fig. 2b) to detect the expected homologous recombination with three primer pairs (primer pairs #4, 5 and 6), to verify that loci were deleted by telomere-associated truncation as expected.

The correct homologous recombination was detected in 10 of 48 (21%) clones. Among them, CHO HisD#44 was negative for all telomere-associated truncation loci (Fig. 3b) (see Supplementary Fig. 1b). Among the 10 clones, which showed positive result with primer pair #3, a morphological change in the size of Chr.21 was observed in four clones (8%) (CHO HisD #04, 21, 36, 44) with more than 25% of 20 analysed cells. FITC signal indicating the presence of transgene was detected on the terminal of the truncated hChr.21 (Fig. 3d). QH banding pattern showed the deletion of long arm of the truncated hChr.21 at q11 to terminal end (Fig. 3e). These PCR and FISH results of telomere-associated truncation suggested that 9 clones except for HisD#44 were a mixture of the truncated and non-truncated cells. MMCT was performed with the four clones (CHO HisD #04, 21, 36, 44) to mouse B16F10 for confirmation of the occurrence of the expected telomere-associated truncation.

### Transfer of the manipulated chromosomes from CHO-K1 cells to recipient cells by MMCT

**Figure 4:**
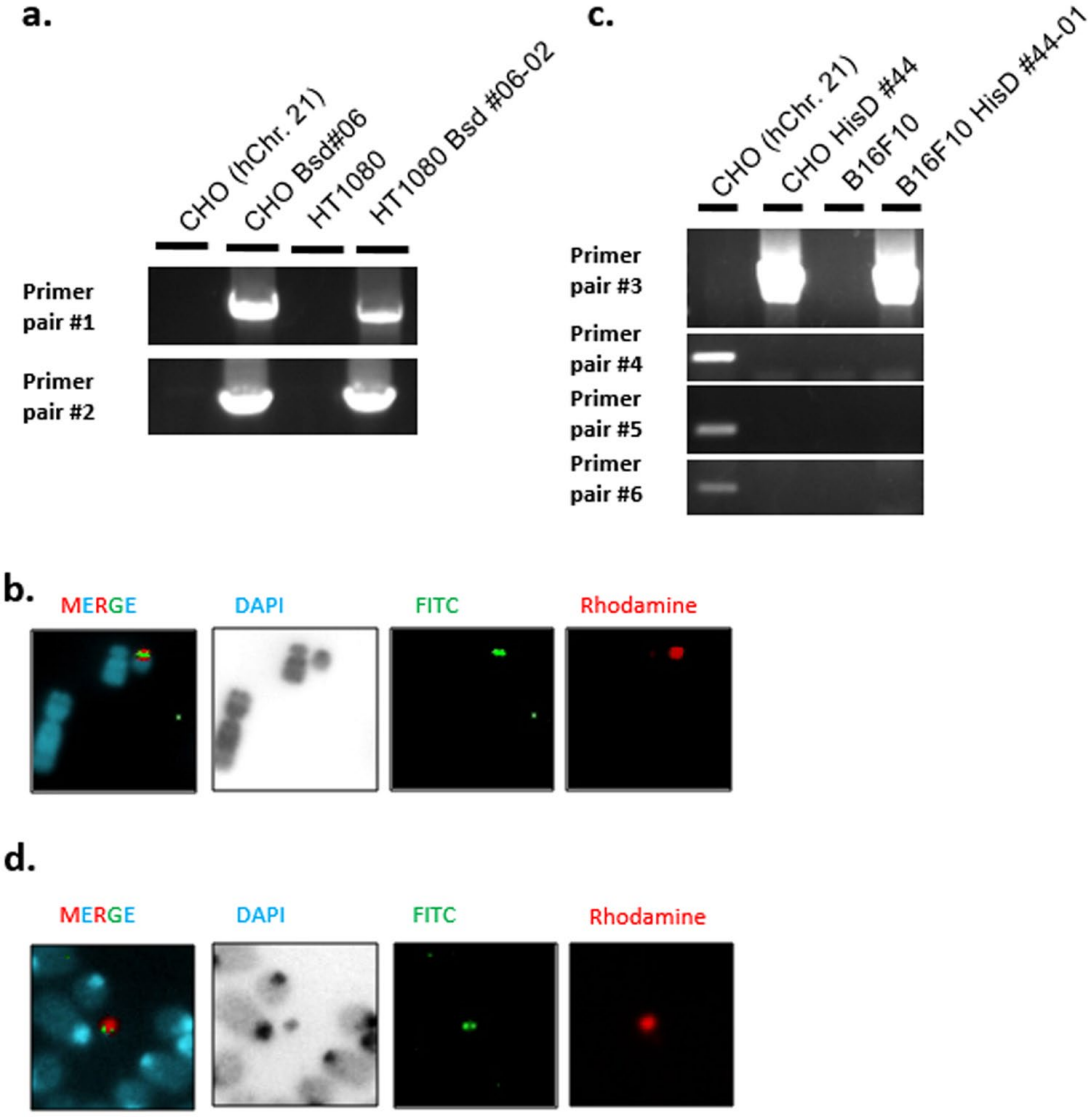
Representative PCR and FISH results of HT1080 and B16F10 cells transferred with the modified hChr.21. (**a**) Representative results of transgene insertion analysed by PCR. (**b**) Representative FISH images of HT1080 Bsd #06-02 transferred with the targeted chromosome. The rhodamine (red) signal indicates the repetitive sequence of plasmid p11-4 containing human alpha-satellite sequences, which stains the centromeric region of human Chr. 21. The FITC signal (green) indicates the presence of the inserted I-EGFP-I-Bsd-Fcy; Fur vector on the near centromeric region. (**c**) Representative results of telomere-associated truncation analysed by PCR. (**d**) Representative FISH images of B16F10 HisD44-01. The rhodamine (red) signal indicates the repetitive sequence of human Cot-1 for staining of whole human chromosome. The FITC signal (green) indicates the presence of pBS-TEL/Dq-HisD vector on the same truncated hChr.21 as observed in the CHO donor cells. Full-length gels of the PCR analysis are presented in Supplementary Figure 2.

To be useful for further biomedical applications, it is essential that the modified chromosomes can undergo MMCT. Five (CHO Bsd #06, 22, 30, 32, 36) clones in which the I-EGFP-I-Bsd-Fcy; Fur vector was inserted, were transferred by MMCT into HT1080 cells as a model of human cancer cells. Thirty-two clones showed well growth and were analysed by PCR. Thirty-one of the 32 clones were positive for the modified hChr.21 by PCR analysis with primer pairs #1 and 2. A representative result is shown in Fig. 4a. For each group derived from each CHO-donor clone, seven PCR-positive-clones were randomly selected for FISH analysis. Six of the 7 clones showed the presence of the expected hChr.21 (Table 2) (see Supplementary Fig. 1a), demonstrating HT1080 clones derived from CHO Bsd clones. Accordingly, 4 of 5 CHO donor clones (CHO Bsd #06, 22, 30, 32) could transfer the expected modified hChr.21. Thus, at least 4 (11%) of 37 isolated CHO clones were confirmed to have the transgene insertion.

**Table 2:** Summary of FISH analysis of the targeted chromosome transferred to HT1080 cells.

Ploidy		2n		
Number of maintained hChr. 21		0	1	2
Clone #	HT1080 Bsd #06-01	12	25	2
	HT1080 Bsd #06-02	8	40	0
	HT1080 Bsd #22-01	10	36	0
	HT1080 Bsd #22-02	12	30	1
	HT1080 Bsd #30-02	4	26	17
	HT1080 Bsd #32-03	6	34	0
	HT1080 Bsd #36-01	40	0	0

Approximately 40 cells were observed to investigate whether the transferred chromosome contained the targeted vector and was maintained independently in HT1080 cells. Each column shows the number of such cells. The ploidy of the HT1080 host chromosomes was 2 n. The numbers of maintained hChr. 21 are shown in the second row.

Four (CHO HisD #04, 21, 36, 44) representative CHO clones containing the truncated human chromosome were used as donor cells to transfer the truncated chromosome to B16F10, because the human chromosome can be distinguished from mouse chromosome. Thus, the truncation can be confirmed. The cells, which were performed MMCT were selected with hisD. Each MMCT experiment produced 3–22 clones. PCR analysis was performed with primer pairs #3, 4, 5 and 6 (Fig. 4c and Table 3) (see Supplementary Fig. 2b). These results revealed that B16F10 clones derived from 3 clones (CHO HisD #04, 36, 44), but not HisD #21, maintained the correct truncated chromosome.

**Table 3:** Summary of PCR results for telomere-associated truncation.

Clone # of CHO donor clone (positive/analyzed)	HisD #04	HisD #21	HisD #36	HisD #44
Primer pair #3	21/22	3/12	2/3	8/8
Primer pair #4	0/22	12/12	2/3	0/8
Primer pair #5	0/22	12/12	0/3	0/8
Primer pair #6	0/22	10/12	0/3	0/8
Correct clone	21/22	0/12	1/3	8/8
(%)	95%	0%	33%	100%

Each column shows the number of the PCR-positive B16F10 clones for each primer pair out of the number of analysed clones. B16F10 clones derived from CHO HisD#4 showed that 21 of 22 were positive with primer pair #3; in contrast, all of the clones were negative with primer pairs #4, 5 and 6. Therefore, 21 of 22 analysed clones (95%) showed an ideal result for PCR, indicating successful truncation of hChr. 21.

FISH analysis confirmed that these PCR-positive B16F10 clones containing the targeted chromosome retained the correct targeted chromosome (Fig. 4d and Table 4).

**Table 4:** Summary of FISH analysis for B16F10 cells transferred with the targeted chromosome.

Ploidy		3n		
Number of maintained hChr. 21		0	1	2
Clone #	B16F10 HisD #04-03	1	19	1
	B16F10 HisD #36-05	1	19	0
	B16F10 HisD #44-02	1	18	1

Approximately 20 cells were observed to investigate whether the transferred chromosome contained the targeted vector and was maintained independently in B16F10 cells. Each column shows the number of such cells. The ploidy of B16F10 host chromosomes was 3 n. The numbers of maintained hChr. 21 are shown in the second row.

Thus, at least 3 (6%) of 48 isolated CHO clones were confirmed to have the telomere-associated truncation.

### The chromosome modification in mouse A9 cells with CRISPR/Cas9

To induce transgene insertion of neomycin resistant gene and EGFP to a hChr.8 in A9 cell^26^ for a model, we designed two sgRNA for CRISPR/Cas9 expression vectors from a region near centromere of the q arm. The A9 cells containing hChr.8: A9 (hChr.8), were electroporated the two CRISPR/Cas9 vectors and the circular targeting vector. Then, 3 clones (43%) among obtained 7 clones showed the occurrence of HR by PCR analysis (see Supplementary Fig. 3a). FISH analysis revealed that 2 clones (29%) (A9 KM47 neo #1, 2) of the 7 clones showed the presence of the transgene on the peri-centromeric region of the q arm on hChr.8 (see Supplementary Fig. 3b). Thus, the chromosome modification techniques with CRISPR/Cas9 was also applicable for A9 human mono-chromosomal library.

## Discussion

In this study, 37 blasticidin-resistant CHO clones were obtained from the transgene insertion experiment, and 17 clones showed correct targeting by PCR analysis. Among them, at least, 5 clones showed expected FISH analysis results, and 4 of the 5 clones could show the transfer of the correct targeted chromosome to HT1080 cells. Thus, at least 11% of the isolated clones contained the correct targeted chromosome and were competent to transfer it to the recipient cells.

For the telomere-associated truncation experiment, 48 blasticidin-resistant CHO clones were obtained, and 10 clones showed correct targeting by PCR analysis, and 1 clone showed perfect PCR results. Among the 10 clones, 5 clones showed expected results for FISH analysis, and 3 of these 5 clones were able to transfer the correctly truncated chromosome to B16F10 cells. Thus, 6% of the isolated clones contained the correctly targeted chromosome and could transfer it to the recipient cell.

For transgene insertion in mouse A9 cells, 7 clones were obtained and 3 of the 7 clones showed correct targeting by PCR analysis. Among the 3 clones, 2 clones showed the expected FISH result. Thus, 29% of the isolated clones contained the correct targeted chromosome.

Thus, we have established a CHO- and A9-mediated chromosome engineering technique, including chromosome transfer directly after chromosome modification in CHO-K1 cells containing a human chromosome.

FISH analysis is necessary to evaluate if a clone has the correct modified chromosome. To obtain a pure population, single cell cloning with drug selection would improve the purity of each CHO donor clone. Scr-7 is an inhibitor of the non-homologous end-joining DNA repair pathway and can increase the efficiency of homologous recombination^29^. In the telomere-associated truncation experiment, the obtained clones were a mixture of the modified and unmodified cells. Therefore, purification of the clones by FACS or limited dilution may be useful to improve this technique.

MMCT has a possibility to induce chromosomal rearrangement. Most recently, to reduce the possibility with reduction of DNA damage, micronucleation via treatment with TN16 and gliseofulvin, and isolation of microcells with latrunculin B instead of colcemid and cytocharasin B^30^ was applied for MMCT as novel methods. The techniques may enable us to obtain the clones without rearrangement of the transferred chromosome.

Thus, the present results advance chromosome engineering for biomedical challenges, such as the production of chromosome aneuploid models and humanized model animals and for identifying genes responsible for genetic disorders^3^.

## Materials and Methods

### Plasmid construction

Original vectors, I-EGFP-I-Bsd, containing HR arms, the blasticidin resistance gene and EGFP, and pBS-TEL/Dq-HisD, containing 1 kb of artificial telomere, an HR arm and a resistance gene against L-histidinol dihydrochrolid, were previously reported^10^. To construct the I-EGFP-I-Bsd-Fcy; Fur vector for transgene insertion, I-EGFP-I-Bsd was digested with SalI and treated with rapid alkaline phosphatase (Roche, Basel, Switzerland). The Fcy; Fur gene was PCR amplified from pSelect-Zeo-Fcy; Fur (Invitrogen, San Diego, USA) and digested with SalI and XhoI. The fragment was then ligated into the SalI-digested I-EGFP-I-Bsd vector. The following primer pair was used to amplify the Fcy; Fur gene: FcyFur EF1 F: 5′-AAAGTCGACAAACAAACTAGCAAAATAGGCTGTCCC-3′ and FcyFur SV40 R: 5′-AAACTCGAGCCATACCACATTTGTAGAGG-3′. pBS-TEL/Dq HisD-Fcy; Fur was constructed in the following way. pBS-TEL/Dq HisD was digested with Srf1 (TOYOBO, Osaka, Japan) and ligated with a double stranded oligo consisting of: AscI-NotI F: 5′-GGCGCGCCTAAGCGGCCGC-3′ and AscI-NotI R: 5′-GCGGCCGCTTAGGCGCGCC-3′. This plasmid was named pBS-TEL/Dq HisD AN. To construct pBS-TEL/Dq HisD Fcy; Fur, pBS-TEL/Dq HisD AN was digested with NotI and treated with rapid alkaline phosphatase, then ligated with an approximately 2.5kb fragment of pSelect-Zeo-Fcy; Fur, digested with EagI. The 1 kb artificial telomere was fragile in certain *E*. *coli* strains; therefore, the pBS-TEL/Dq HisD series was introduced by electroporation and amplified in DH10B (Thermo Fisher, Waltham, USA), an *E*. *Coli* strain that could stably maintain the 1 kb artificial telomere. The length of the artificial telomere was confirmed by digestion with HindIII, producing an approximately 1.4 kb band.

### CRISPR/Cas9 design

Genomic sequence data of AP000167 and AP001657 loci were obtained from the NCBI database (Accession #. NC_000021 GPC_000001313).

I-EGFP-I-Bsd contained a left arm (approximately 3.4 kb), whose sequence was between 5′-GGGCCCTTAGTGAGAGTTTG-3′ and 5′-ATAGAAGTCCAGGCTGTGGGGCCC-3′ and a right arm (approximately. 2.4 kb)^10^, whose sequence was between 5′-TCTAGAAACTGAATTTATAG-3′ and 5′-GTTACCCAGGATGATCTAGA-3′. The targeted sequences for the CRISPR/Cas9 vector were selected from the sequences between the left and right arm. The telomere truncation vector, pBS-TEL/Dq HisD, contained a homologous recombination arm (approximately 7kb), whose sequence was between 5′-GGATCCTGAGGCCTGGCAGCGGGCGC-3′ and 5′-TACAATAGAAACCAGAATAA-3′. Therefore, the targeted sequences for CRISPR/Cas9 were downstream of the homologous recombination arm. The CRISPR/Cas9 targeted sequences were designed by the web site, Optimized CRISPR design tool (http://crispr.mit.edu/)^26^. The DNA oligomer in below, was inserted into the BbsI site of pX330-U6-Chimeric_BB-CBh-hSpCas9, which was a gift from Feng Zhang (Massachusetts Institute of Technology) (Addgene plasmid # 42230).

AP000167T1, targeting 5′-GTAAGCCACAAGACAAGTCAGG-3′ was constructed with 5′-CACCGGTAAGCCACAAGACAAGTC-3′ and 5′-AAACGACTTGTCTTGTGGCTTACC-3′. AP000167T11, targeting 5′- GAAGAAACATGGCCTAAGGCAGG-3′ was constructed with 5′-CACCGGAAGAAACATGGCCTAAGGC-3′ and 5′-AAACGCCTTAGGCCATGTTTCTTCC-3′.AP001657T5, targeting 5′-GAGAATTGCTTGAACCAGGGAGG-3′ was constructed with 5′-CACCGGAGAATTGCTTGAACCAGGG-3′ and 5′-AAACCCCTGGTTCAAGCAATTCTCC-3′.AP001657T T6, targeting 5′-CTCCTGAGCAGCTGGGACGCAGG-3′ was constructed with 5′-CACCGCTCCTGAGCAGCTGGGACGC-3′ and 5′-AAACGCGTCCCAGCTGCTCAGGAGC-3′.

### Evaluation of Cleavage activity for the CRISPR/Cas9 vectors by Cel-I assay

Surveyor® Mutation Detection kit (Integrated Device technology, San Jose, USA) was used for evaluation of cleavage activity of the designed CRISPR/Cas9 expression vectors. Each expression vector was transfected into HEK293FT cells (Thermo Fisher), which were seeded at 500,000 cells per well in a 12-well plate. The transfection was performed with Lipofectamine LTX (Thermo Fisher) and 1µg of each plasmid vector, following the manufacturer’s instructions. Forty-eight hours after transfection, genomic DNA of the transfected cells was extracted using a Gentra Puregene cell kit (Qiagen, Germantown, USA). The following primer pairs were used for detection of cleavage activity. AP001657T4-7 loci F1:5′-ACAATAGAAACCAGAATAAAAATGG-3′ and AP001657T4-7 loci R1:5′-ATGGTCCATGGGGTTTATCC-3′ were used for evaluation of cleavage activity of AP001657T5 and AP001657T6 CRISPR/Cas9 vectors. AP00167F2:5′-TGTGACCACCATTGACACCTG-3′ and AP00167R2: 5′-CTACAGGCTCCCACCACCAC-3′were used to evaluate the cleavage activity of AP000167T1 CRISPR/Cas9 vector. AP00167T8-12 loci F1: 5′-TGCCTCTTCTGTGGTGAGTG-3′ and AP00167T8-12 loci R1: 5′-ACTGCATTTGTGTCCCTGTG-3′ were used to evaluate the cleavage activity of AP000167T11 CRISPR/Cas9 vectors. The PCR amplification of the targeted regions was performed with ExTaq (TakaraBio, Kusatsu, Japan). The amplified PCR products were re-annealed by being heated to 95 °C for 10 min, then cooled from 95 °C to 85 °C over 5 sec, from 85 °C to 25 °C over 600 sec, followed by incubation at 25 °C for 1 min, and holding at 4 °C.

Finally, surveyor nuclease treatment was performed under the following conditions. Ten µl of the PCR product, 1 µl SURVEYOR enhancer S, 1 µl SURVEYOR Nuclease S and 0.15 M MgCl_2_ were mixed, and incubated at 42 °C for 60 min. Then, the mixture was separated by electrophoresis in a 3% agarose gel and stained with ethidium bromide. Gels were recorded using a gel imaging apparatus (ATTO Technology, Getzvill, USA). The pictures were analysed by image J and a previously reported method^31^.

### Transfection for transgene insertion and telomere-associated truncation

pBS-TEL/Dq HisD-Fcy; Fur was linearised with NotI and purified by ethanol precipitation and then used for the following transfection. CHO (Chr.21) cells were transfected with 1.1 µg of targeting vector or telomere-associated truncation vector and 1.5 µg of each of the two CRISPR/Cas9 expression plasmid vectors. Lipofection was performed with Lipofectamine 2000 (Thermo Fisher) following the manufacturer’s instructions. Five days after transfection, cells were expanded to five10-cmdishes, and appropriate selectable antibiotics added: 8 µg/mL blasticidin S (Sigma-Aldrich, St. Louis, USA) or 7 mM L-histidinol dihydrochrolid (Sigma-Aldrich) for positive selection of the recombined cells, and 200 µM flucytosine (5-FC) (Sigma-Aldrich) for negative selection of unexpected insertion of the plasmid vector containing Fcy; Fur.

### Cell culture

CHO-K1 and CHO (Chr.21) containing human chromosome 21^10^ were cultured in Ham’s F12 (Wako, Osaka, Japan) containing 10% fetal bovine serum (FBS)(Biowest, Vieux Bourg, France), 1% penicillin /streptomycin (Wako) and 800µg/mL G418 (Promega, Madison, USA). HT1080 and B16F10 cells were cultured in Dulbecco′s Modified Eagle’s Medium (DMEM) (Wako) containing 10% FBS.

### PCR

Primer sequences are described below. Primer pair #1 was I-EGFP-I-Bsd long F1: 5′-CCTGATTGCCCTGGCCAGAACTTCCATCAC-3′ andI-EGFP-I-Bsd long R1: 5′-CGCCCTCTCGCACGATTACCATAAAAGGCA-3′, primer pair #2 was Bsd F: 5′-CAACAGCATCCCCATCTCTG-3′ and#21CenG3R: 5′-TTTAGATGCAGGGGCATACTGTGAGCAT-3′, primer pair #3 was q2L:5′-TCATGCCACAATCAATCTCCCAAGTAGC-3′ and sk23: 5′-GGCCGCTCTAGAACTAGTGGATC-3′, primer pair #4 was Chr.21 15F (SHGC-10662-F): 5′-TTTTGTCTTAGGATTAGACGTGACC-3′, Chr.21 15R(SHGC-10662-R): 5′-AGAACTGGGAAGTCTCATAACTGG-3′, primer pair #5 was Chr.21 34F (PCP4(WI-14954)-F): 5′-CCTTGTAGGAAGGTATAGACAATGG-3′ and Chr.21 34R (PCP4(WI-14954)-R): 5′-GAATTCACTCATCGTAACTTCATTT-3′ and primer pair #6 was Chr.21 49F (SIM2(SIM2)-F): 5′-AAAGCCAACAAACCAAGAC-3′ and Chr.21 49R (SIM2(SIM2)-R): 5′-TTGTAGCAAACACGAGCC-3′.

### FISH

Metaphase chromosomes were prepared from colcemid-treated cell cultures by hypotonic treatment with 0.075 M KCl and methanol/acetate (3:1) (Wako) fixation. FISH was carried out using human Cot-1 DNA for analysis of CHO and B16F10 or p11-4^32^ plasmid containing alpha satellite sequence of Chr.13 and21 for HT1080, labelled with digoxigenin (Roche) and PCR product, amplified from I-EGFP-I-Bsd with primers 5′-GCAACGTGCTGGTTATTGTG-3′ and 5′-AAGGAAAAGTTTAAACTTAGCCCTCCCACACATAAC-3′, and PCR product from pBS-TEL/Dq-HisD with primers 5′-TCGAGGTGAGCCCCACGTTCTG-3′ and 5′-TCGAGGTGAGCCCCACGTTCTG-3′, labelled with biotin (Roche). The DNA probes were labelled with a nick translation kit (Roche). The digoxigenin-labelled DNA probes were detected with an anti-digoxigenin-rhodamine complex (Roche), and the biotin-labelled DNA was detected using avidin-conjugated fluorescein isothiocyanate (FITC; Roche). The chromosomes were counterstained with 4′,6-diamidino-2-phenylindole (DAPI)(Sigma-Aldrich). Metaphase images were captured digitally with a CoolCubeI CCD camera mounted on a fluorescence microscope (Axio Imager, Z2; Carl Zeiss, Promenade, Jena, Germany). Images were processed using the ISIS software provided with the microscope^17^.

### Quinacrine hoechst staining

Slides of metaphase spreads were stained with quinacrine mustard (Sigma-Aldrich) and Hoechst 33258 (Sigma-Aldrich) for observation of the chromosome size and banding pattern. Metaphase images were captured digitally with a CoolCubeI CCD camera mounted on a fluorescence microscope were processed using the ISIS software provided with the microscope.

### MMCT

Donor CHO cells were expanded in T-25 flasks (Thermo Fisher), in Ham’s-F12medium containing 20% FBS and 0.1 µg/mL colcemid (Thermo Fisher) and incubated at 37 °C for 48h to induce micronucleation. The culture medium was then refreshed, and cells were incubated for another 24 h. The T-25 flask containing CHO cells with micronuclei was filled with DMEM containing 10 µg/mL cytochalasin B (Sigma-Aldrich) and centrifuged for 1h using an Avanti HP-26XP, JLA-10,500 rotor (Beckman Coulter Life Sciences, Indianapolis, USA) at 11,900 × g to form microcells. The pellet including microcells was collected and filtered through 8-, 5-, and 3-µm pore-size filters to purify microcells. Microcell pellets were collected by centrifugation at 760 × g with a table-top centrifuge (Kubota Corporation, Tokyo, Japan). To introduce the chromosome fragments to the HT1080 or B16F10 recipient cells, 2 × 10^6^ cells were prepared in a 6-cm dish (Corning, Corning, USA). The microcell pellets were suspended in 2mL DMEM containing 0.05 mg/mL phytohemagglutinin P (Sigma-Aldrich). This suspension was added to the recipient cells and the dish was incubated for 20 min and then treated with PEG1000 (Wako) containing 10% DMSO to fuse microcells and recipient cells. These fused cells were incubated for 24 h at 37 °C in DMEM containing 10% FBS and 12 µg/mL of blasticidin S for HT1080 cells and 7 mM L-histidinol dihydrochrolid for B16F10 cells^33^.

## Electronic supplementary material

Supplementary Figure
