# Supplementary material for: CRISPR/Cas9-induced transgene insertion and telomere-associated truncation of a single human chromosome for chromosome engineering in CHO and A9 cells
Source: Sci Rep. 2017 Oct 6;7:12739. doi: 10.1038/s41598-017-10418-7 (PMC5630592; doi:10.1038/s41598-017-10418-7)
Supplement: Supplementary file 1 — Supplementary Figure [file 41598_2017_10418_MOESM1_ESM.doc]

**CRISPR/Cas9-induced transgene insertion and telomere-associated truncation of a single human chromosome for chromosome engineering in CHO and A9 cells.**

Narumi Uno^1,2^, Kei Hiramatsu^1^, Katsuhiro Uno^1^, Shinya Komoto^1^, Yasuhiro Kazuki^1,2,3^, Mitsuo Oshimura^2^,*

^1^Department of Biomedical Science, Institute of Regenerative Medicine and Biofunction, Graduate School of Medical Science, Tottori University, 86 Nishi-cho, Yonago, Tottori 683-8503, Japan

^2^Chromosome Engineering Research Center, Tottori University, 86 Nishi-cho, Yonago, Tottori 683-8503, Japan

^3^Department of Molecular and Cellular Biology, Faculty of Medicine, Tottori University, 86 Nishi-cho, Yonago, Tottori 683-8503, Japan

*Corresponding author: oshimura@med.tottori-u.ac.jp


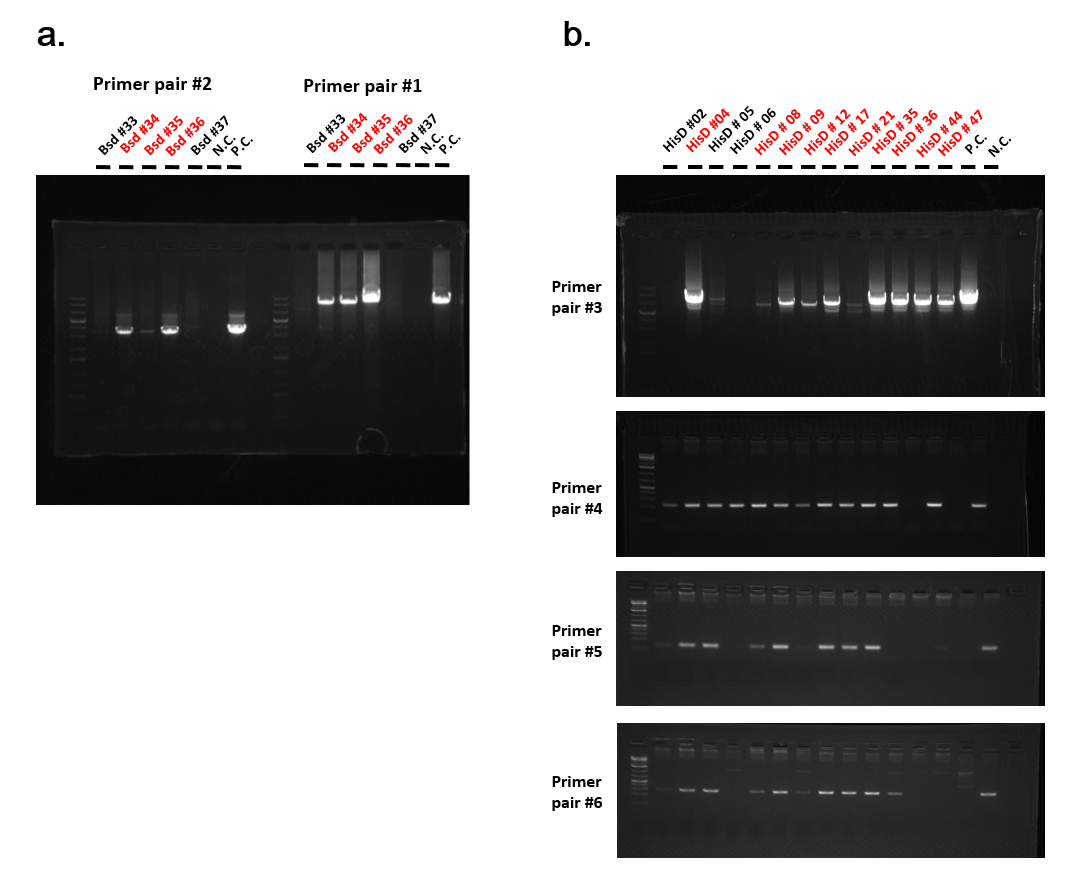


**Supplementary Figure 1. Full-length gels of PCR analysis for the transgene insertion and the telomere-associated truncation in CHO cells.**

a). Representative results of transgene insertion analysed by PCR (red for positive clones). b). Representative results of telomere-associated truncation analysed by PCR (red for positive clones with primer pair #3).


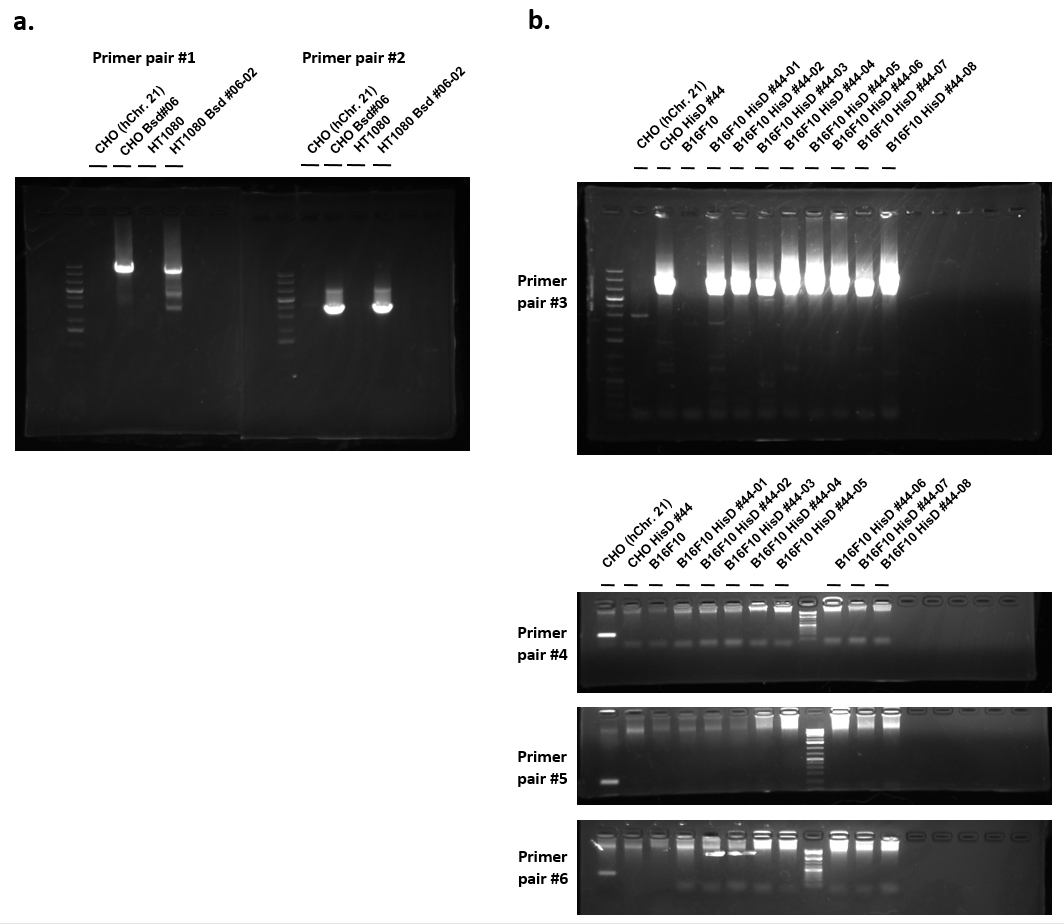


**Supplementary Figure 2. Full-length gels of PCR analysis for the modified chromosome in HT1080 and B16F10 cells.**

a). Representative results of PCR analysis for HT1080 cells, transferred the modified hChr.21 with transgene insertion. b). Representative results of PCR analysis for B16F10 cells, transferred the modified hChr.21 with telomere-associated truncation.


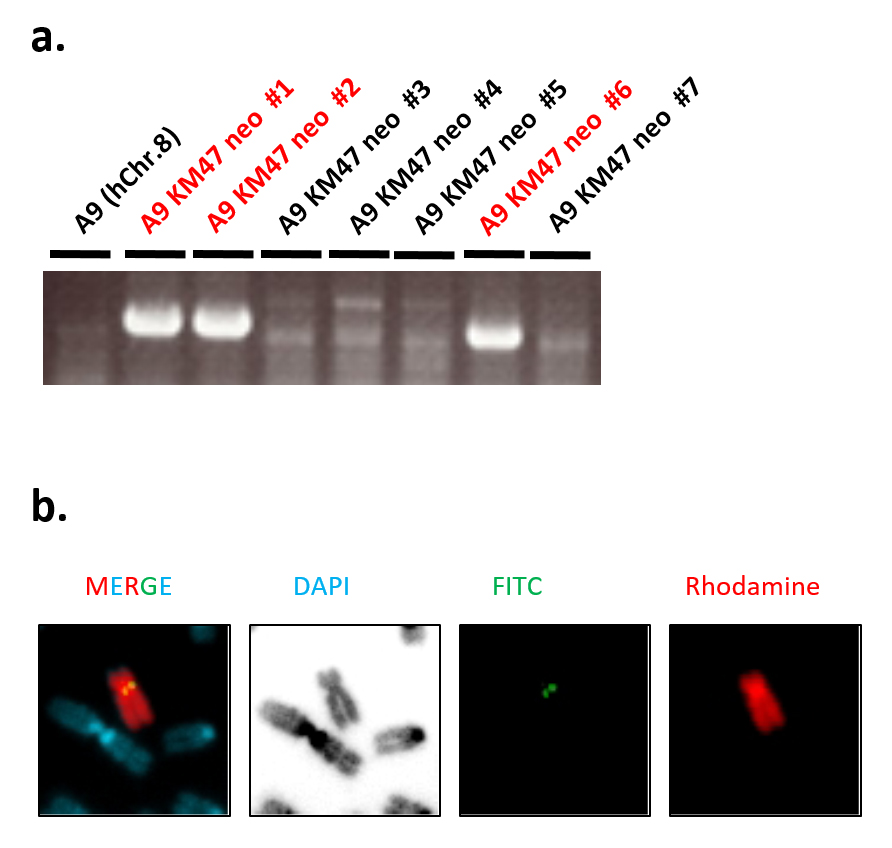


**Supplementary Figure 3. Representative PCR and FISH results of mouse A9 cells containing the modified hChr.8.**

a). Representative results of transgene insertion analysed by PCR (red for positive). The negative control was A9 (hChr.8), which contained an original human chromosome 8. b). Representative FISH images of transgene insertion. The rhodamine (red) signal indicates the repetitive sequence of human Cot-1 for detection of whole human chromosome. The FITC signal (green) indicates the inserted targeting vector in the centromeric region of hChr.8.
